# Denosumab in osteoarthritis: from mechanism to clinical translation

**DOI:** 10.3389/fimmu.2026.1822628

**Published:** 2026-05-25

**Authors:** Jiaoniu Duan, Gailian Zhang, Liyun Zhang

**Affiliations:** 1 Third Hospital of Shanxi Medical University, Shanxi Bethune Hospital, Shanxi Academy of Medical Sciences, Tongji Shanxi Hospital, Taiyuan, China; 2 Department of Rheumatology and Immunology, Fifth Hospital of Shanxi Medical University, Shanxi Provincial People’s Hospital, Taiyuan, China

**Keywords:** cartilage degeneration, denosumab, fibroblast-like synoviocytes, FSTL1, macrophage, osteoarthritis, RANKL, synovial inflammation

## Introduction

Osteoarthritis (OA) is the most common joint disorder, affecting over 600 million people worldwide (1). Despite this immense burden, no disease-modifying osteoarthritis drug (DMOAD) has been approved to date that can alter the course of joint pathology (2). Consequently, the age at which patients undergo total joint replacement is decreasing, further amplifying the societal and economic burden of OA (3).

Prevention and early intervention remain critical to reducing this burden—yet current treatment is initiated only after symptoms are established and relies on modalities with limited efficacy, as underscored by numerous phase II and III randomized controlled trials (RCTs) that have shown only modest or no clinical benefits (2). This therapeutic stagnation has prompted a fundamental re-evaluation of OA pathogenesis. A paradigm shift is now reframing OA from a localized “wear-and-tear” condition to a systemic disease driven by metabolic, inflammatory, and ageing-related factors (3). This new understanding opens unprecedented opportunities for therapeutic intervention, including the repurposing of existing biologics.

Denosumab, a human monoclonal antibody targeting receptor activator of nuclear factor kappa-B ligand (RANKL) widely used in osteoporosis, has garnered attention for its potential efficacy in OA based on accumulating preclinical and clinical evidence. This has sparked interest in repurposing it as a DMOAD—yet critical questions remain: Which patients should receive denosumab? What is the optimal dose? How should discontinuation be managed? And can findings from hand and knee studies be extrapolated to other joints? This Opinion addresses these questions with specific evidence and proposes a research agenda to guide clinical translation.

## Mechanistic expansion

Shangguan and colleagues demonstrated that, in human chondrocytes and osteoclast precursors *in vitro*, denosumab blocks RANKL-induced osteoclastogenesis via nuclear factor kappa-B (NF-κB) inhibition while attenuating ROS-induced chondrocyte apoptosis, with corresponding upregulation of anabolic genes (SOX9, COL2A1) and suppression of hypertrophic markers (COL10A1, RUNX2) via TGFβ1/smad3 signaling (4).

Synovial inflammation is now recognized as a key driver of OA pathogenesis, with imaging studies demonstrating its presence in both early and advanced-stage disease (5). Using single-cell RNA sequencing of murine knee joints with post-traumatic OA, Hu and colleagues identified fibroblast-like synoviocytes (FLSs) as the primary source of RANKL in the OA joint (6). Mechanistically, RANKL signals through RANK on synovial macrophages and FLSs, activating NF-κB signaling that drives transcription of pro-inflammatory mediators including tumor necrosis factor (TNF), interleukin (IL)-6, C-C motif chemokine ligand 2 (CCL2), and matrix metalloproteinases (MMPs). In FLSs, this transcriptional program also induces follistatin-like protein 1 (FSTL1), an NF-κB target gene. Secreted FSTL1 acts as a pro-inflammatory mediator that activates neighboring macrophages, establishing a forward amplification loop (6). Denosumab disrupts this pathological circuit by blocking RANK/TNF receptor-associated factor 6 (TRAF6)/NF-κB-dependent FSTL1 induction in FLSs, thereby preventing FLS-mediated paracrine activation of macrophages and targeting both the stromal and myeloid compartments (6).

**Figure 1:**
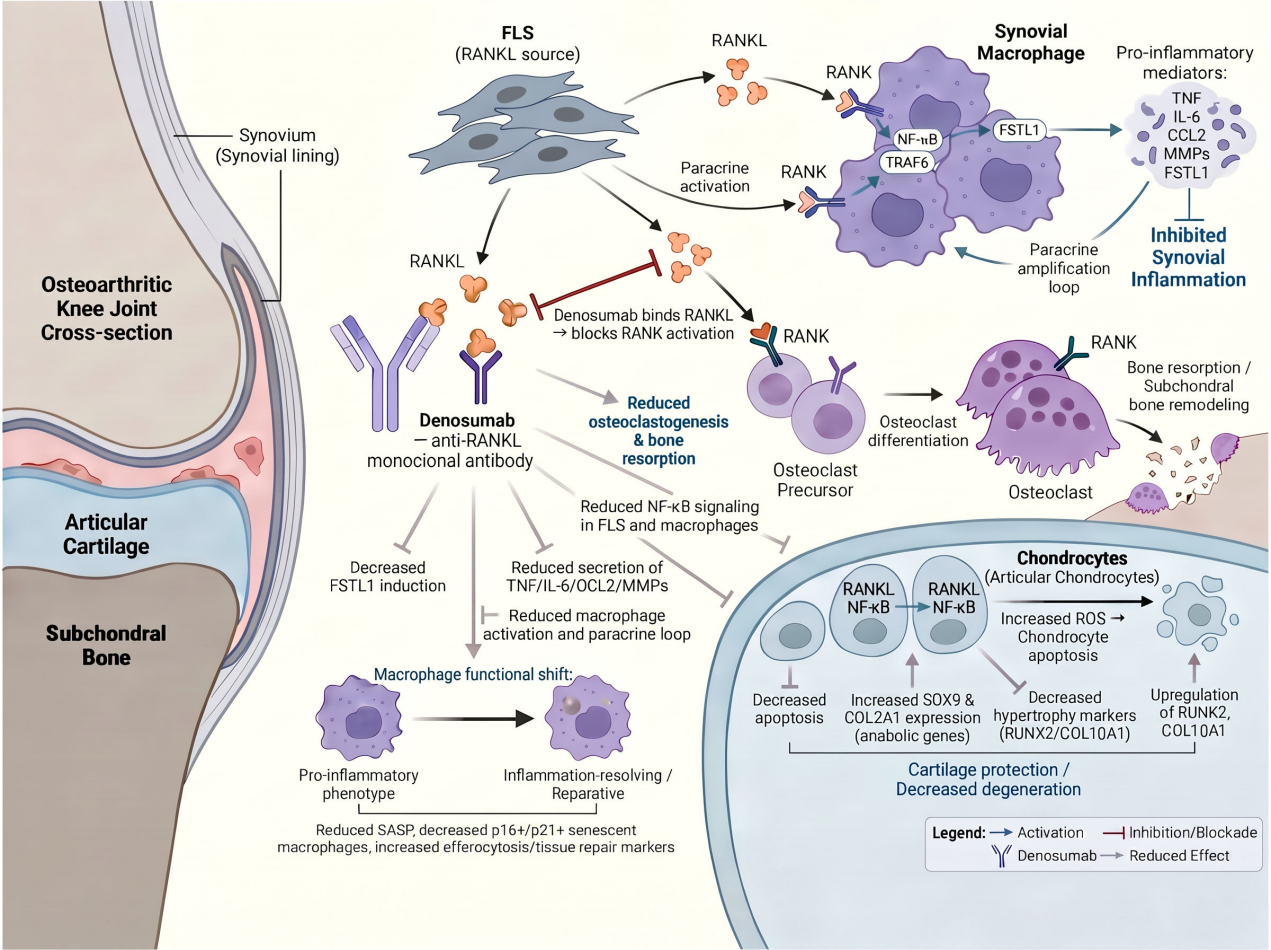
Mechanism of action of denosumab in OA. Denosumab, by binding and neutralizing RANKL, disrupts synovial fibroblast-macrophage crosstalk, inhibits osteoclastogenesis and bone resorption, shifts synovial macrophages toward a reparative phenotype, and protects chondrocytes through reduced NF-κB signaling and enhanced anabolic gene expression. Collectively, these effects attenuate synovitis, cartilage degeneration, and abnormal subchondral bone remodeling in OA.

Macrophages are central to OA pathogenesis, with their functional state critically influencing disease progression (7). Single-cell transcriptomic studies have revealed multiple functionally distinct macrophage subpopulations in human OA synovium, spanning a continuous spectrum from pro-inflammatory and catabolic to inflammation-resolving and reparative (7). In a rodent model of OA, Ming and colleagues showed that locally delivered denosumab attenuates synovial macrophage senescence, reducing senescence-associated secretory phenotype (SASP) gene expression and p16^+^/p21^+^ macrophage abundance (8). In naturally aged mice, Liao and colleagues demonstrated that macrophage-targeted denosumab shifts the activation profile toward an inflammation-resolving state, characterized by reduced pro-inflammatory mediators and increased markers of efferocytosis and tissue repair, while improving Osteoarthritis Research Society International (OARSI) scores and bone mineral density (BMD) (9). The proposed mechanisms of denosumab in the OA joint are summarized in **Figure 1**.

These mechanistic findings should be interpreted with caution. The molecular pathways described above have been elucidated primarily in animal models and *in vitro* systems, and the translational gap between preclinical OA models and human disease remains substantial. Confirmation of these mechanisms in well-phenotyped OA patient cohorts is therefore needed.

## Clinical evidence

The therapeutic potential of denosumab in OA was first evaluated in a single-center, randomized, double-blind, placebo-controlled phase 2a trial in erosive hand OA (10). Wittoek and colleagues enrolled 100 patients (mean age 61 years, 78% female) with at least one interphalangeal joint in the erosive or joint space loss phase and ultrasonographic evidence of active synovitis. Patients were randomized 1:1 to denosumab 60 mg subcutaneously every 3 months or placebo for 48 weeks, followed by a 48-week open-label extension. The intensified dosing—double the standard osteoporosis regimen—was informed by dose-dependent structural inhibitory effects of denosumab observed in rheumatoid arthritis (10). The primary endpoint, change in Ghent University Scoring System (GUSS) score at week 24, showed a between-group difference of 8.9 points (95% CI 1.0–16.9; p = 0.024), widening to 14.3 points (95% CI 4.6–24.0; p = 0.003) at week 48. Denosumab also reduced new erosive joints by 76% (OR 0.24, 95% CI 0.08–0.72; p = 0.009). Clinical benefits emerged in the extension phase, with significant improvements in pain and function at 96 weeks (10). Regarding safety, adverse events occurred in 80% of denosumab-treated patients versus 90% with placebo; infections (51% vs. 45%) and musculoskeletal symptoms (41% vs. 49%) were most common. Six serious adverse events were reported in the denosumab group, with no new safety signals identified. However, the long-term safety of this intensified regimen in a non-osteoporotic population remains to be established, and these findings require confirmation in larger multicenter studies.

Following these advances in hand OA, attention has extended to knee OA. A single-arm study in 9 patients observed improvements in magnetic resonance imaging (MRI) synovitis scores, Oxford Knee Score, and painvisual analogue scale (VAS) pain following denosumab treatment (6). Given the small sample size and uncontrolled design, these findings should be viewed as hypothesis-generating only.

Larger-scale real-world evidence provides complementary but similarly preliminary support. A Taiwanese cohort study of over 13,000 women with concomitant knee OA and osteoporosis found that denosumab users had a 23% lower risk of total knee arthroplasty compared with bisphosphonate users (aHR 0.77, 95% CI 0.62–0.97; p = 0.024), with the strongest effect in patients aged ≥80 years (aHR 0.39, 95% CI 0.20–0.77; p = 0.007) (11). Although propensity-score matching was employed to balance the groups, the database lacked key variables including body mass index, BMD, baseline knee OA severity, and sociodemographic factors such as income and education. As matching can only adjust for measured confounders, these unmeasured differences may partly account for the observed reduction in knee arthroplasty risk, and the retrospective design precludes causal inference.

A TriNetX analysis of over 118,000 adults with osteoporosis followed for up to 10 years demonstrated a 13% lower risk of incident knee OA among denosumab users (HR 0.87, 95% CI 0.84–0.91), with a more pronounced association in Asian participants (HR 0.73, 95% CI 0.63–0.84) (12). Subgroup analyses revealed consistent associations in females (HR 0.87) and adults aged ≥65 years (HR 0.88). No significant association was observed for hip OA (HR 0.98, 95% CI 0.94–1.03), highlighting a critical evidence gap (12). However, generalizability may be limited by the predominantly non-Hispanic White cohort with relatively high socioeconomic status. Residual confounding by indication remains possible, as longitudinal weight data were inconsistently captured and other unmeasured variables could not be adjusted for despite propensity-score matching. These observational findings should therefore be viewed as hypothesis-generating.

Collectively, these clinical findings generate a consistent but preliminary signal across hand and knee OA, spanning RCT and real-world study designs. The convergence of structure-modifying efficacy in a controlled trial of erosive hand OA and reduced knee OA progression in observational cohorts supports the rationale for dedicated RCTs to establish causality.

## Delivery innovations

Systemic denosumab faces inherent limitations for localized joint disease, including suboptimal bioavailability and infection risks with intra-articular injections. Two recent studies have addressed these challenges through innovative delivery platforms.

Ming and colleagues developed dissolvable microneedle patches loaded with denosumab (MNs@De) for transcutaneous intra-articular delivery (8). In rodent and canine OA models, MNs@De achieved sustained drug retention for seven days, significantly reducing Mankin scores from 10.24 to 5.03 (p < 0.001)—an effect comparable to intra-articular injection and superior to systemic administration (8).

Liao and colleagues employed folate-modified liposomes (DS@Lip-FA) for targeted macrophage delivery, achieving significantly higher accumulation in synovial macrophages than non-targeted liposomes (9). This approach effectively reversed the age-related M1/M2 imbalance, promoting an anti-inflammatory macrophage phenotype while improving OARSI scores and bone mineral density in aged mice (9).

Together, these delivery innovations address key limitations of systemic administration by enhancing local bioavailability, prolonging drug retention, and enabling cell-specific targeting.

## Unanswered questions

Despite substantial progress, several critical questions must be addressed to translate these findings into clinical practice.

Which phenotypes respond? OA is a heterogeneous syndrome for which multiple classification approaches have been proposed, including stratification by molecular, clinical, and imaging features. The Rapid Osteoarthritis MRI Eligibility Score (ROAMES) categorizes knee OA into inflammatory, subchondral bone, meniscus/cartilage, atrophic, and hypertrophic structural subtypes based on MRI features (13). Whether certain subtypes respond preferentially to denosumab remains an open question. From a mechanistic standpoint, the inflammatory subtype—characterized by synovitis and effusion—involves the FLS-macrophage crosstalk that denosumab disrupts, while the subchondral bone subtype reflects high-turnover osteoclast activity that RANKL inhibition could theoretically attenuate. These mechanistic considerations provide a rationale for investigating differential treatment effects across subtypes, but prospective phenotype-stratified trials are needed to determine whether such differences exist and which classification approach best predicts treatment response.

Beyond structural phenotypes, demographic factors may also influence treatment response. A stronger association was observed in Asian participants (12), with similar patterns in females and older adults (11, 12). The reasons for these stratified associations remain unknown. While genetic background, environmental exposures, or epigenetic modifications could theoretically modulate RANKL pathway activity and denosumab responsiveness (14), direct evidence supporting any specific mechanism is lacking. Future studies incorporating pharmacogenomic and proteomic analyses may help identify predictive biomarkers and elucidate whether these differential associations reflect underlying biological differences or residual confounding.

What is the optimal dose? The hand OA trial used 60 mg every three months—double the osteoporosis dose (10). Real-world knee OA studies used standard dosing and still showed risk reduction (11, 12), suggesting standard dosing may suffice for prevention while intensification may be needed for treatment. Pharmacokinetic/pharmacodynamic studies with serial biomarker measurements (synovial RANKL, FSTL1) are needed to establish dose-response relationships.

How to manage discontinuation? In osteoporosis, denosumab discontinuation triggers rebound bone turnover—C-terminal telopeptide of type I collagen (CTX) increases up to 70% above baseline, BMD returns to pretreatment levels within 12–24 months, and vertebral fracture risk rises (15). Similar skeletal risks must be anticipated in OA patients, many of whom have concomitant age-related bone loss. Beyond skeletal concerns, whether rebound bone turnover could exacerbate subchondral bone pathology or accelerate cartilage degeneration remains unknown. Furthermore, the rapid restoration of soluble RANKL upon discontinuation could reactivate RANK-expressing synovial macrophages and FLSs, potentially triggering a rebound of joint inflammation—a phenomenon that has not been studied but warrants consideration given the inflammatory component of OA. This creates a distinct clinical dilemma. In osteoporosis, transitioning to bisphosphonates after denosumab discontinuation effectively preserves bone mass. However, bisphosphonates act by inducing osteoclast apoptosis and do not interrupt RANKL-mediated signaling in synovial macrophages or FLSs; they are therefore unlikely to replicate denosumab’s joint-protective effects. For an OA patient without a compelling osteoporosis indication, this raises a difficult scenario: bisphosphonate therapy may be required, not for its direct benefit, but solely to mitigate the skeletal consequences of withdrawing a drug given for joint disease. Future OA trials must pre-specify exit strategies evaluating both skeletal and joint-specific outcomes—including structural MRI and patient-reported symptoms—across extended follow-up (16).

## Discussion

Is denosumab a DMOAD for OA? Based on current evidence, the answer is not a simple yes or no, but rather phenotype-dependent and joint-specific.

For erosive hand OA, this trial provides encouraging proof-of-concept evidence. The 14.3-point improvement in GUSS score at 48 weeks and 76% reduction in new erosive joint development (10) are clinically meaningful effect sizes. However, the single-center design and modest sample size limit generalizability, and these findings should be viewed as preliminary rather than definitive. A larger, multicenter phase 3 trial is now warranted to confirm these results and support regulatory approval.

For knee OA, the evidence is encouraging but incomplete. Real-world studies offer two complementary perspectives: denosumab is associated with a reduced risk of incident knee OA in osteoporosis populations—suggesting a preventive effect—and with a lower likelihood of total knee arthroplasty in patients with established disease, indicating potential disease-modifying activity (11, 12). These epidemiological signals are consistent with preliminary observations of improved synovitis in a small single-arm study (6). Collectively, these findings—spanning both primary prevention and secondary modification of disease progression—underscore the need for confirmation in dedicated RCTs.

For hip OA, we face a critical evidence gap. The null finding from real-world data (HR 0.98, 95% CI 0.94-1.03) neither confirms nor refutes efficacy, as the confidence interval cannot exclude clinically meaningful benefit or harm (12). Dedicated hip OA studies, whether prospective cohorts or RCTs, are therefore warranted to address this uncertainty.

Mechanistically, denosumab exerts pleiotropic effects across multiple joint tissues—inhibiting osteoclast-mediated bone resorption, suppressing FLS activation, and shifting synovial macrophages toward an inflammation-resolving state (4, 6, 8, 9). This mechanistic breadth is conceptually attractive for a heterogeneous disease like OA. Yet mechanism does not guarantee clinical benefit across all phenotypes. The translation from molecular effect to patient outcome depends critically on which pathological driver predominates in a given individual—underscoring the imperative for phenotype-driven research to identify those most likely to benefit.

Critically, the unanswered questions are now well-defined and actionable. First, phenotype-driven RCTs with baseline ROAMES and biobanking must identify which structural subtypes derive greatest benefit. Such studies should also incorporate multi-omics analyses to explore the biological basis of differential responses by ethnicity, sex, and age. Second, pharmacokinetic/pharmacodynamic studies with serial biomarker measurements must establish optimal dosing. Third, the long-term safety of denosumab in OA populations requires systematic evaluation. While osteoporosis experience has established a decade-long safety profile, OA trials may involve higher doses, different dosing intervals, or longer treatment durations. Future trials must therefore include comprehensive safety surveillance throughout the treatment period, with particular attention to rare adverse events such as atypical femoral fractures and osteonecrosis of the jaw. Fourth, dedicated hip OA studies must address the current evidence vacuum.

In summary, whether denosumab can become a viable DMOAD depends on the rigor of future trials designed to address the outstanding questions outlined above. The field now has the tools to conduct such trials—the priority is to use them.
